# Hypodontia and Delayed Dentition as the Primary Manifestation of Cleidocranial Dysplasia Presenting with a Diagnostic Dilemma

**DOI:** 10.1155/2012/262043

**Published:** 2012-12-25

**Authors:** Radhika Chopra, Mohita Marwaha, Payal Chaudhuri, Kalpana Bansal, Saurabh Chopra

**Affiliations:** ^1^Department of Pedodontics and Preventive Dentistry, SGT Dental College & Research Institute, Budhera 123505, Gurgaon, Haryana, India; ^2^Department of Pediatrics, Subharti Medical College, Meerut 250005, India

## Abstract

Cleidocranial dysplasia is a rare autosomal disorder which manifests as partial or complete absence of clavicles, multiple supernumerary teeth, and delayed closure of fontanelle. Classical cases of cleidocranial dysplasia are easily diagnosed very early in the life. However, cases with partial manifestation of the syndrome and noncontributory family history are difficult to diagnose. Here, we report a case of 8.5-year-old girl child who presented with delayed tooth development (without any supernumerary teeth), anterior open fontanelle, and normal clavicles, thus resulting in a diagnostic dilemma.

## 1. Introduction

Cleidocranial dysplasia (CCD) is a dominant, inherited autosomal bone disorder with a wide range of expressivity, primarily affecting bones undergoing intramembranous ossification and characterized by clavicular aplasia or hypoplasia, retarded cranial ossification, supernumerary teeth, short stature, and a variety of other skeletal abnormalities [1].

The classical features of this syndrome are partial or complete absence of the clavicles, multiple supernumerary teeth [2], and delayed closure of the sagittal fontanelle [3]. Other features include a bell-shaped thorax, enlargement of frontal and occipital bones, hypoplasia of the pelvis and distal phalanges, short stature, hypertelorism [4], and impacted permanent teeth. Less common findings of CCD patients include shortened or absent nasal bones, reduced or absent paranasal sinuses, thickening of some segments of the calvaria, underdevelopment of maxilla, and delayed union of mandibular symphysis [5]. 

Here, we report a case of 8.5-year-old female child who did not manifest the classical features of CCD, thus resulting in diagnostic dilemma.

## 2. Case Report

A 8.5-year-old girl was referred to the department of pedodontics and preventive dentistry with the chief complaint of delayed eruption of permanent teeth. She was born prematurely at 8 months to healthy parents, and her birth weight was 2.9 kg. The family history was unremarkable.

**Figure 1:**
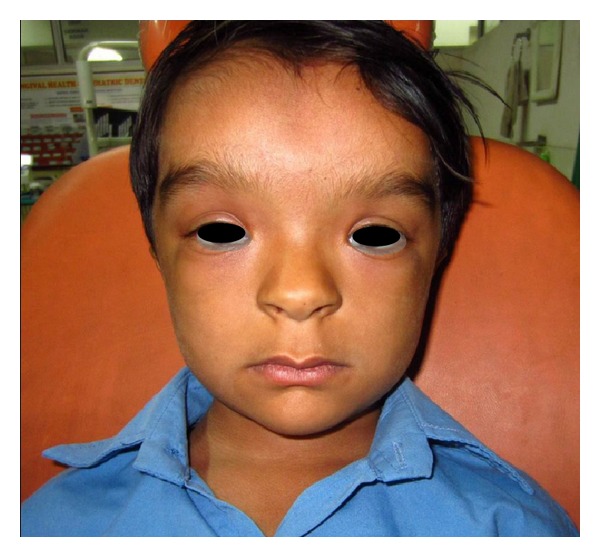
Facial photograph.

**Figure 2:**
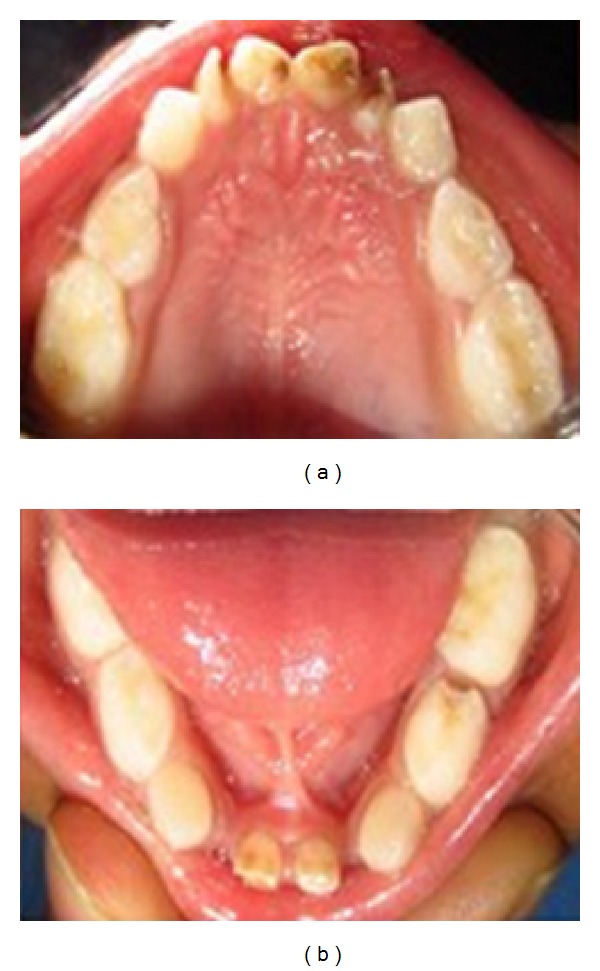
Intraoral photograph.

General examination of the patient revealed a weight of 22 kgs, height of 109 cms, and head circumference of 53 cm. On extraoral examination, the patient presented with frontal bosselation, hypertelorism, epicanthal fold with respect to left eye, and depressed nasal bridge (Figure 1). Open fontanelle could be palpated in the anterior region of the head. Intraoral examination revealed the presence of a set of deciduous dentition with missing primary mandibular right and left lateral incisors and none of the permanent teeth had erupted as yet (Figure 2).

**Figure 3:**
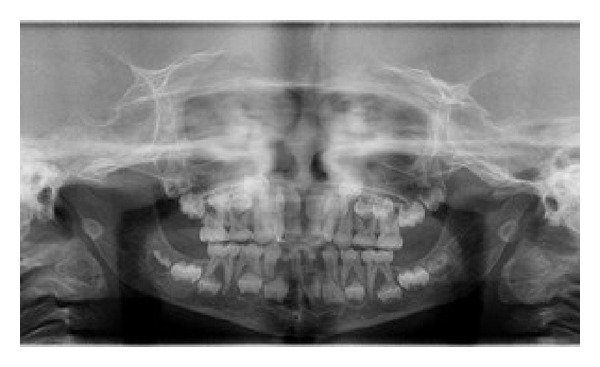
OPG.

**Figure 4:**
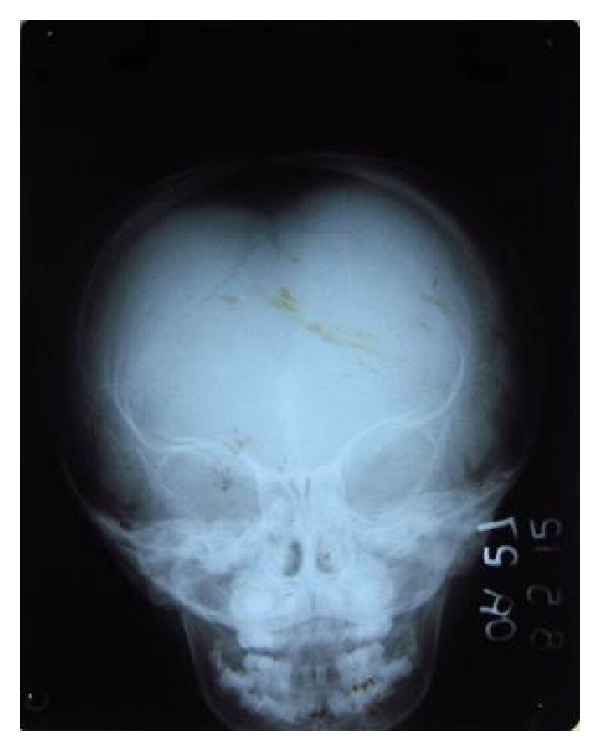
PA view skull.

**Figure 5:**
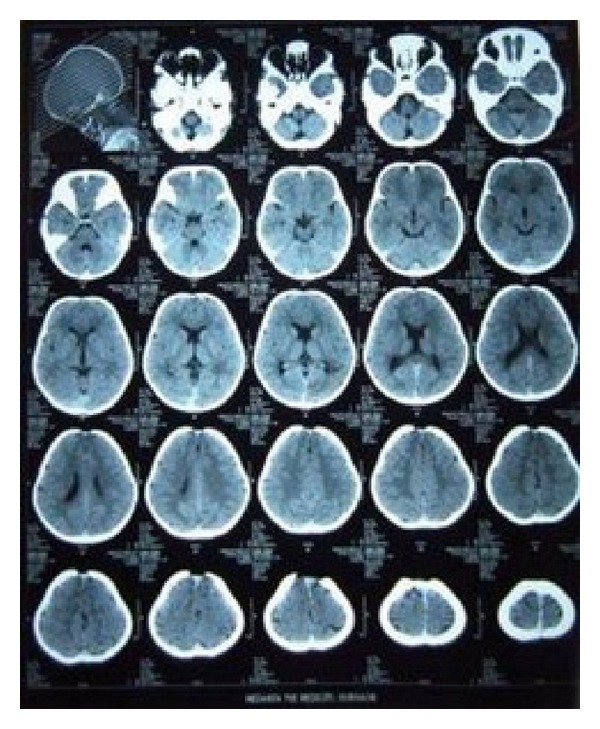
CT scan skull.

An orthopantomogram revealed absence of permanent tooth buds of permanent mandibular right and left lateral incisors and permanent mandibular right central incisor (Figure 3) and there was a generalized delay in the development of permanent tooth buds. Delayed root formation of permanent first molars was noted. No supernumerary teeth were seen in the radiograph. PA skull revealed delayed closure of fontanelle (Figure 4). CT scan of skull was done revealing focal defect in frontal bone measuring upto 3.8 cm (Figure 5).

**Figure 6:**
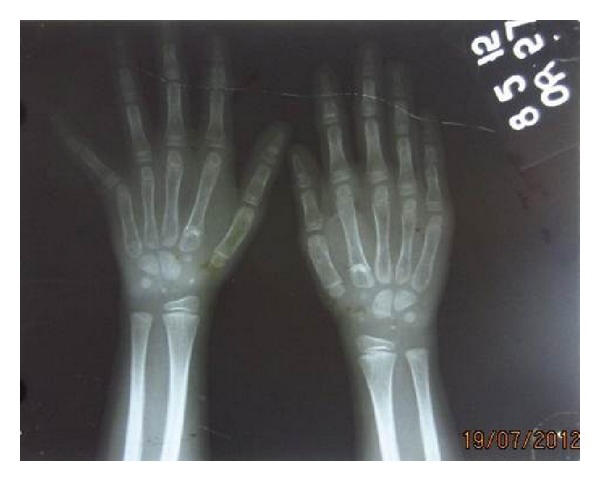
Hand wrist radiograph.

The posteroanterior view of chest radiograph showed normally developed clavicles. Hand wrist radiograph showed short distal phalanges and the bone age was found to be on the lower limit for her age (Figure 6). Thyroid function test was also normal.

So the prominent findings in our case were delayed development of permanent dentition, frontal bossing, and anterior open fontanelle. These findings did not correlate with any other syndrome except for CCD.

**Figure 7:**
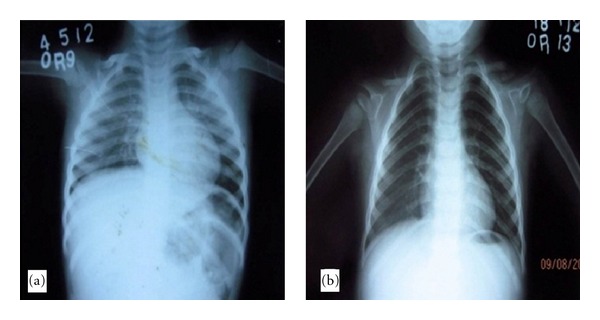
Chest radiograph: (a) patient and (b) sister.

Thereafter, all the family members were screened for any manifestation of CCD. Only the younger sister showed hypermobility of the shoulders. Complete physical and radiologic assessment revealed abnormal clavicles with a small right clavicle and two pieces of left clavicle (Figure 7). There was no other dental or skeletal abnormality detected. 

Based on this finding, a diagnosis of cleidocranial dysplasia was made. The decayed anterior teeth of the patient were restored and the patient is being kept under regular followup to evaluate the eruption of permanent teeth. The orthodontic and prosthodontic intervention will be planned as required.

## 3. Discussion 

CCD was first described by Pierre Marie and Paul Sainton in 1898 [6]. CCD is also known as Marie-Sainton disease, mutational dysostosis, and cleidocranial dysostosis. This condition is usually caused by a mutation of the Core binding factor alpha 1 (Cbfa1) gene, located at chromosome 6p21, which is essential for osteoblasts and odontoblasts differentiation as well as for bone and tooth formation [7]. 

CCD is a relatively uncommon disorder with a prevalence of 0.5 per 100,000 live births [8]. Clinically, the diagnosis is often made at birth but may not occur until later, when persistence of the widely open anterior fontanelles and sutures or short stature incites parental concern. Individuals with this disorder present with some or all of very characteristic features. Skeletal abnormalities commonly found include clavicular aplasia/hypoplasia, bell-shaped thorax, enlarged calvaria with frontal bossing and open fontanelles, Wormian bones, brachydactyly with hypoplastic distal phalanges, hypoplasia of the pelvis with widened symphysis pubis, severe dental anomalies, and short stature. Our patient showed no pathology in the clavicular and pelvic bones but there was a presence of open fontanelle, frontal bossing, and hypoplastic distal phalanges. Clavicles are underdeveloped to varying degrees in these patients and are completely absent in approximately 10 percent [1, 9, 10]. Patients with normal clavicles have also been described in previous studies [11]. 

Dental changes occur frequently and are very characteristic of CCD. The large number of supernumerary teeth that form a more or less complete third dentition (up to 30 extra teeth in some cases) is one of the most striking findings in CCD. Contrary to this, delayed eruption without supernumerary teeth was present in our case which made it difficult to reach a definite diagnosis, as such findings are also frequently observed in other conditions such as osteopetrosis or pycnodysostosis. CCD is also associated with a delay of root development in permanent dentition and a lessened but not entirely absent eruptive potential [12]. These features were in accordance with the findings in our case. 

In CCD, many of the deciduous teeth are retained throughout life and lie among the permanent teeth. The permanent teeth generally lose their eruption stimulus and stay embedded, while the deciduous teeth are retained. Suggested factors for overretained deciduous teeth are lack of eruption potential and lack of cellular cementum on roots of permanent teeth, delayed mineralization of teeth, physical barrier-abnormal density of bone overlying the succedaneous teeth, and failure of bony crypt to resorb [13]. In our patient there was delayed eruption of permanent teeth and tooth buds of 32, 41, 42 were missing. 

CCD involves mutation in the transcription factor, Runx2/Cbfa1, located on chromosome 6p21 [7]. There is a notably phenotypic variation of CCD even within one and the same family. In approximately 40% of CCD patients, a genetic transition cannot be identified, and the condition develops spontaneously [5, 14]. In our report, both the sisters were affected with CCD with variable manifestations. The patient showed hypodontia and delayed permanent dentition, open fontanelle with normal clavicles while the sister had only defective clavicles without any other manifestations of CCD. 

Osseous development is severely delayed in the newborn period and manifested by the delayed ossification of skull and the pubis, absence of nasal bones, and the incomplete fusion of the vertebral arches [4, 15]. The abnormal modeling of bone is manifested in the calvaria, by delayed ossification, causing secondary ossification centres to form and the bones of the skull to be thin and with abnormal margins. This causes the sutures to be widely spaced and late closing [4, 15]. Our patient showed a large defect in the frontal bone measuring upto 3.8 cms. 

The planning of treatment for patient with CCD is complicated by a number of factors and largely depends on both chronological and dental ages of the patient. The timing of diagnosis is not only important for choosing an appropriate treatment plan but also for obtaining successful treatment results. A team approach to management of dental abnormalities on a long-term basis is necessary. The overall goal is to provide an aesthetic facial appearance and functional occlusion by late adolescence or early adulthood.
